# Trends in Physical Activity and Motor Development in Young People—Decline or Improvement? A Review

**DOI:** 10.3390/children11030298

**Published:** 2024-03-01

**Authors:** Cristiana D’Anna, Pasqualina Forte, Elisa Pugliese

**Affiliations:** 1 Department of Psychology and Education, Pegaso University, 80143 Napoli, Italy; pasqualina.forte@unicam.it (P.F.); elisa.pugliese@unicam.it (E.P.); 2 School of Advanced Studies, University of Camerino, 62032 Camerino, Italy

**Keywords:** motor skills, motor performance, sedentary lifestyle, trends, youth

## Abstract

This paper aims to analyse studies and research conducted in EU and non-EU member states to identify and compare trends in physical activity and motor skills. Thus, a comparative overview of the state of the art in the two pre-pandemic decades that can facilitate an understanding of the different territorial realities and training needs to be conducted, considering the different cultural situations. A scoping review was conducted by analysing a series of studies published between 1990 and 2022, including only those studies that collected data in the pre-pandemic period. The search was carried out on databases such as PubMed, Google Scholar, and ScienceDirect. The following keywords were used for the acquisition of relevant studies: children, decline, motor skills, physical activity, and young people. From the literature review emerged clearly in all the longitudinal surveys a negative trend of body mass index (BMI), which was increasing in all the countries analysed, and cardiorespiratory fitness, which, in close relation to the increase in overweight levels, was in decline. If an unambiguous trend could be declared for the variables just mentioned, it was not possible to declare the same trend for the other variables analysed, which showed discordant results between the different countries.

## 1. Introduction

Consistent physical activity offers lifelong advantages for health. Among children and adolescents, engaging in physical activity enhances various aspects such as musculoskeletal and cardiometabolic health, cognitive growth, motor abilities, self-confidence, social interaction, academic achievement, and overall welfare [1]. Stressing the pivotal role of physical activity in promoting health and holistic well-being, the Physical Activity Guidelines advocate for children and young adults to participate in at least sixty minutes of moderate to vigorous physical activity daily. However, it is concerning that many teenagers fail to meet this recommendation. According to the results from the international survey, Health Behaviour in School-aged Children, only 25.0 % of boys and 15.0 % of girls claim to meet the amount of physical activity suggested by the WHO [2].

Despite the emphasis placed on the benefits of physical activity in promotion guidelines, there is a noticeable decline in participation in physical and sports activities as time progresses. These guidelines aim to acknowledge and encourage the significance of maintaining healthy levels of physical activity and minimising sedentary behaviour across various educational settings, both formal and informal.

In the formal context such as the school, there is a heterogeneous trend between the different states in the organisation of the teaching of physical education.

It is necessary for educational institutions and researchers to carefully verify which teaching methodologies and approaches are particularly effective in promoting participation in physical and sports activities from an inclusive perspective. These approaches should encourage the actively engaged participation of students in meaningful and challenging experiences, allowing them to explore, experiment, and develop their skills [3].

The correlation between physical education and social dynamics is complex, albeit not always immediately apparent. Motor and sports sciences are instrumental in the growth of youth and the acquisition of essential cross-cutting skills beneficial for everyday life and social cohesion. Participation in physical activity and sports can function as a valuable resource, offering a premium experience that fosters an educational journey [4].

A 2019 study that compared school curricula across European countries highlighted that the duration allocated to physical education in certain EU member states falls short of meeting the recommendations set by the WHO. In some instances, physical education assumed a peripheral role in the school curriculum, indicating not only a deficiency in action but also underscoring the necessity to champion innovative and effective policy solutions. These solutions could serve as models for other countries that are presently less inclined to prioritise investments in both the quality and quantity of physical education within both school and extracurricular settings [5]. 

A sedentary lifestyle, often associated with inadequate nutrition, is a global public health problem, with a high disease burden and associated social costs [6]. The decrease in the physical activity of children and teenagers has a direct impact on overweight and obesity and leads to a worsening of motor performance, physical efficiency indices, and the capability of carrying out daily work with rigour and ability, with the negative consequences at the cardiovascular and metabolic levels [7,8,9].

Children’s engagement in physical activity could potentially influence the development of their motor skill proficiency. Higher levels of physical activity offer increased chances to stimulate neuromotor development, consequently fostering the development of fundamental motor skills (FMSs) [10]. In general, young children exhibit diverse levels of motor skill competence largely due to variations in their experiences. These differences stem from numerous factors such as their immediate surroundings, access to structured physical education, socioeconomic status, parental guidance, climate, and more [11,12]. Family socialisation and the example set by parents regarding health-related behaviours play a pivotal role in shaping children’s own health-related behaviours [13]. Moreover, excessive sedentary screen time, particularly television (TV)-related screen time, is the primary contributor to overall inactivity among children and adolescents. In fact, some have proposed that the association between screen time and obesity might hold limited clinical significance in children and youth [14,15].

Furthermore, the acquisition of fundamental movement skills holds particular significance for preschoolers as it can determine whether children possess the necessary skills to progress to a more specialised movement phase in sports [16]. 

Identifying trends (levels of physical and sedentary activity) in children and teenagers is important for understanding changes in population health, possible political interventions, investments, and progress towards what may be national and international goals [17]. This is also important because the levels of participation of young people in physical activity and sports are often influenced by the quality of the proposals within the different learning contexts, first and foremost, the school environment.

Supporting the fact that physical activity brings benefits not only on a physical level but also on a mental one in all age groups, especially in children, scholars offer considerable insights. The scientific literature suggests that physical activity in children has a positive effect on cognition and academic performance [18].

Chaput et al. [19] confirm that greater amounts and intensities of physical exercise have positive effects on health, especially at the cardiorespiratory, muscular, bone, and metabolism levels. Therefore, the higher the frequency of physical activity, or the less sedentary the lifestyle, the better the quality of life related to health [20].

This paper aims to analyse and compare studies and research conducted in EU and non-EU member states to compare trends in motor skills and competencies in young people, identify general trend lines (decline or improvement) if existing for the different analysed variables, and provide a comparative framework summarising the state of the art in the two pre-pandemic decades, which can facilitate an understanding of the different territorial realities and what training needs are emerging, considering the different cultural situations that exist. A useful framework to guide policy choices and investments and enable different stakeholders to design and implement improvement actions is proposed.

## 2. Materials and Methods

### 2.1. Data Sources and Search Strategies

A scoping review was conducted by analysing a series of studies published between 1990 and 2022, including only those studies that collected data in the pre-pandemic period. A scoping review serves as a method to summarise the existing knowledge surrounding a particular subject by examining a collection of literature. It brings attention to a range of studies and scientific findings pertinent to the subject matter [21].

This review was performed in accordance with PRISMA guidelines [22]. The search was carried out on databases such as PubMed, Google Scholar, and ScienceDirect. The following keywords were used for the acquisition of relevant studies: trend, decline, physical activity, motor skills, children, and young people. The selection criteria identified studies focusing on physical activity and movement, obesity levels, motor skills development, psychosocial development, and other health indicators.

### 2.2. Eligibility Criteria

Studies that met the following inclusion criteria related to the PICOS (participants–interventions–outcomes–study design) strategy were included in this review [22]. 

Specifically, the following criteria were defined: age range of the survey sample 6–18 years; studies with at least 1000 subjects involved; no experimentation; and assessment of levels and/or trends in motor skill levels. 

The search was conducted by two reviewers, who selected the studies separately according to criteria defined ex ante by the analysis of the titles and abstracts. Subsequently, all authors independently studied the selected abstracts to determine their final eligibility according to the exclusion and inclusion criteria. The references of the selected studies were reviewed to find further studies for inclusion in this research.

Only longitudinal studies published in peer-reviewed journals written in English or Italian language were considered. 

### 2.3. Study Procedures

In the first phase, a selection of titles was made; then, the abstracts were examined for suitability. In addition to examining the full text of the abstracts that met the criteria, all full texts whose abstracts did not provide sufficient information for eligibility were also analysed.

**Figure 1:**
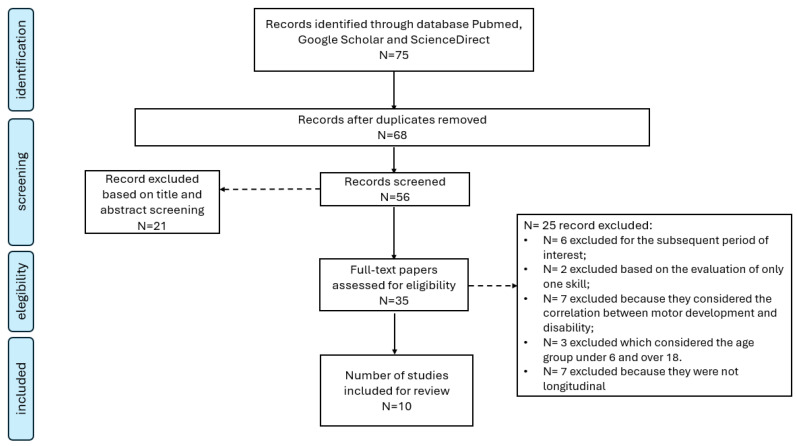
Study flow diagram.

Figure 1 illustrates the information flow throughout various stages of the literature review, encompassing the identification of the literature, screening of studies, assessment of eligibility, and inclusion of final articles. Initially, the search yielded seventy-five potentially pertinent studies. Subsequently, after removing duplicates and screening titles and abstracts, thirty-five full-text articles were retrieved. Among these articles, ten studies fulfilled the inclusion criteria (Table 1). These studies were conducted in Italy, China, Canada, Portugal, the USA, Croatia, Slovenia, the Czech Republic, Korea, and Lithuania.

In order to provide a synthetic framework that differentiated positive from negative trends, the studies were entered into the table by specifying whether the survey results showed a declining trend (ꜛ) or, on the contrary, an improving trend (ꜜ).

## 3. Results

Data from each included study were processed and inserted into Table 1, divided by significant items. The items in the table were divided as follows: source (author, year of publication), title, sample of the study (sample size, period, age of the participants), objective of the study, methodology of the research, main results, country in which the research was carried out, examined trends, and whether they improved (ꜛ) or declined (ꜜ).

A summary framework (Table 1) emerged from the report, in which it was possible to trace the selected studies from EU and non-EU member states, with an immediate comparison at the international level. 

In order to be able to best identify the general trends of the individual variables and/or macro-areas investigated in the different European and non-European countries, a summary report was produced which, from the interpretation of the specific results that emerged in the various longitudinal studies conducted, highlighted the definition of general trend lines (decline or improvement) where they existed for the different variables and then described the data of the variables analysed, highlighting differences and similarities.

The studies analysed investigated a wide range of variables related to participation in physical activity and physical performance levels. Comparisons were, therefore, only possible for the same variables examined over the same age range. The choice to investigate specific variables was certainly also influenced by cultural aspects and the specific emergencies of each country. 

What emerged clearly was the general deterioration of the body mass index (BMI) value in Portugal, the USA, Croatia, Slovenia, the Czech Republic, and Korea; in all the countries in which this variable was analysed, there was a longitudinal worsening trend. 

Cardiorespiratory fitness was evaluated in the age range of 8 to 18 years showing, in line with the BMI data, a general tendency to worsen: the levels of aerobic resistance and aerobic performance tested in Canada, Italy, Croatia, Slovenia, Korea, and Lutuania declined. 

In the data on aerobic endurance capacity, compared to the general upward trend in BMI, a clear decline was identified in the various studies examined; the examination of the other variables analysed did not show any points of convergence that would allow similar trends to be assembled and identified.

The analysis of the co-ordination variable, a skill that was tested longitudinally only in Croatia (11–14 years), where the data were positive for girls but negative for boys, and in Italy (6–13 years), where the recorded trend worsened, did not allow similarities to be found.

Similarly, the flexibility variable showed the same levels or a slight improvement over the years in Canada (6–10 years); in Croatia (11–14), it was improved in girls as opposed to boys, in which it worsened; in Portugal (10–11 years) and Lithuania (11–18 years), the analysis shows a worsening trend. This, as pointed out, was evaluated for different age ranges, so they are certainly not comparable data. 

Upper body strength (including the core) improved in Portugal and Croatia while it worsened in Lithuania; lower body strength was positively evaluated in Croatia, Portugal recorded an improvement in sprint time but jumping performance remained unchanged, and Lithuania reported a worsening of this parameter.

The measurement of daily minutes dedicated to physical activity was tracked only in the USA (11–16), where the trend was positive, as opposed to in the Czech Republic (14–18) where there was a decline.

Quite different was the Chinese Longitudinal Survey, in which a general decline in physical fitness was recorded, assessed through a battery of tests oriented towards the measurement of speed, endurance, and strength in the different muscle districts. This trend, in contrast to the Eastern Front, showed an improvement in Croatia. 

## 4. Discussion

Understanding general trends regarding participation in physical activity and levels of motor performance is essential to acknowledge the changes that are occurring, as the evolution and development of motor skills are fundamental indicators of health in young people [33,34,35,36,37].

These data, closely connected to the conditions of obesity and overweight in developmental children and teenagers, show a negative effect on the development of motor skills, physical activity levels, and psycho-affective factors [38,39,40,41,42,43].

The research by Ortega et al. [34], who subjected young adolescents to a battery of motor tests on conditional and coordinative skills, shows that the level of physical efficiency is an important indicator of health for children and young people and is closely related to abdominal adiposity. Indeed, the study showed that improving muscular efficiency and quickness, rather than cardiorespiratory capacity, appears to have positive effects on humour and self-confidence and appears to be associated with good school performance as well as improving skeletal development.

According to this perspective, it becomes essential to counteract as much as possible the tendency to reduce physical activity, which, especially in industrialised countries, is a phenomenon that no longer affects only the adult population but extends to young people, starting from pre-school age. 

Table 1 summarises the studies that conducted longitudinal analyses to highlight long-term trends in the levels of participation in physical activity, body mass index, and levels of motor skills and abilities in younger generations between 6 and 18 years of age. In order to provide an overview of the different trends, improving trends (ꜛ) were identified on the one hand and trends showing declining trends (ꜜ) were identified on the other hand, specifying the variables in question. The following discussion intends to focus on these two blocks, already reported and described in different or similar trends in results, to critically analyse the similarities and differences that characterise the trends of physical activity and physical fitness in the youth population in relation to the cultural characteristics of the different territorial contexts and the existing literature. 

An Italian study recorded alarming secular trends of involution of physical efficiency in the paediatric age. A decline in motor skills, in particular, coordination, was found in an Italian regional sample of over 1000 students attending secondary school during over years in 1999–2004. In fact, the cross-sectional study confirmed the hypothesis of a secular involutional trend in aerobic endurance associated with an involution of rapid force control coordinative performance, indicative of a decrease in force endurance already within a time interval of a few tens of seconds. This secular involution seems to affect the natural longitudinal development of performance during the developmental age. The only performances that improved in these fifteen years were those of oculo-manual coordination and fast and explosive strength with high technical content; this was probably due to the strong prevalence of introductory sports content in physical education teaching practices in the Italian secondary school context. In this regard, the study emphasises the need to promote the development of a broad base of physical and coordinative efficiency, optimally developed to ensure the further growth of motor performance over time [26]. Only recently (from 2022/2023 school year) has the Ministry of Education introduced specialised teachers in primary schools; previously, physical education was conducted by generalist teachers. This condition has over the years affected both the general physical fitness levels of Italian children and education on healthy lifestyles. Several studies, in fact, confirming previous annual reports, show from the longitudinal perspective that the percentage of overweight children is 20.4%, obese children account for 9.4%, and 2.4% are severely obese, placing Italy among the European countries with the highest values of excess weight among the school-age population. There is a clear geographical trend that sees the southern regions having higher values of excess weight in all genders. Higher prevalences of obesity are also observed in more disadvantaged socioeconomic families. The indicators referring to physical activity and movement were almost stable over the years, indicating that there is still much to be done in terms of promoting these healthy lifestyles [44].

Not surprisingly, a study conducted by Monacis et al. [45] to evaluate the progression of muscle strength in adolescents from a region of Apulia, Italy, compared motor performance between 1990 and 2020. The findings revealed concerning data indicating not only a significant increase in the percentage of overweight and obese male adolescents, rising from 5.3% in 1990 to 33.3% in 2020, and from 10.0% to 47.2% for females, but also a decline in motor performance from 1990 to 2020 for both boys and girls, irrespective of BMI status.

In Slovenia, a study aimed to estimate trends in children’s physical fitness. During the whole study period, 1989–2019, physical fitness showed negative trends, with some exceptions: positive trends were observed in the neuromuscular aspects of physical fitness, particularly in females. Up until the past ten years, cardiorespiratory fitness declined in all age groups, with the biggest declines happening before the year 2000. The last ten years saw a change in trends. The flexibility indicator showed the greatest disparities between genders, with girls experiencing primarily positive trends and boys experiencing mostly negative ones [29]. This finding confirms that of an earlier Slovenian study that highlighted a significant reduction in the strength levels of the lower and upper limbs, with peaks (in terms of involution) observed in the years 1993/1994–2003/2004, thus emphasising the existence of a negative trend between the increase in anthropometric factors (in particular BMI) and the decline in motor skills [46]. 

This was also shown by a study by Sirressi and Colella [47], which analysed the levels of motor performance, lower and upper limb rapid strength, speed, and motor coordination in relation to BMI, also taking into account gender differences. The study evaluated the hypothesis that overweight subjects have lower levels of motor performance than their normal-weight peers, especially in tests involving body movement. The authors assessed motor skills using different motor tests such as long jump from a standing position, seated frontal throw of basketball, 10 m and 20 m fast run, and 20 m slalom dribbling with basketball. These data represent the entire paediatric age group (2–18 years), allowing international comparisons. The study showed significant differences by sex and group regarding performance in quick strength, speed, and motor coordination. Overweight conditions negatively affected motor development and motor learning processes.

The study confirms previous scientific evidence showing how overweight and obesity, in addition to being health risk factors, affect motor performance and developmental milestones. The negative relationship between motor performance and BMI is demonstrated by many works in the literature. A systematic review conducted in 2022 [48] demonstrates through the analysis of 33 scientific studies that children and adolescents with higher BMIs have lower levels of moderate to vigorous physical activity, as well as lower levels of gross motor coordination. So, this relationship affects both coordinative and conditional motor skills, resulting in reduced aerobic capacity, speed, or lower limb strength, and this is negatively reflected in the performance of locomotor skills. Fundamental motor skills such as running, throwing, catching, kicking, and hitting are inversely related to BMI and waist circumference, making overweight individuals less skilled.

The trend of increasing BMI and the resulting decline in motor skills is also recorded by a study conducted in Lithuania. The survey conducted between 1992 and 2012 presents the largest ever data on changes in physical fitness for both sexes of schoolchildren aged 11–18. The study used a Eurofit test battery to evaluate balance, flexibility, muscle strength and power, agility, and cardiorespiratory fitness and, through anthropometric measurements, the body mass index (BMI) of the subjects was calculated. While there were improvements in girls’ abdominal muscle strength, boys’ agility, and both sexes’ balance over the same period, the results showed a loss of flexibility, reduced cardiorespiratory fitness, and reductions in lower limb muscle power and upper body strength. Agility and abdominal muscle strength showed growing patterns prior to 2002, but, between that year and 2012, they began to decline. While all groups’ BMIs increased, balance kept getting better [32].

Regarding the marked decrease in aerobic activity, fitness, and flexibility and slight increase in muscular endurance among adolescents aged 12, 14, and 16 years, an earlier 10-year study compared data collected in Lithuania in 2002 with data from 1992. The researchers hypothesised that one of the causes of this decline could be attributed to the slow progress of physical education reform in schools during Lithuania’s first decade of independence, in which slow changes in the physical education curriculum contributed to arousing little interest among students, resulting in low motivation for physical activity [49].

In Portugal, a study analysed trends in the anthropometry and physical fitness of children between 1993 and 2013, emphasising the increasing weight gain compared to height in boys while, in girls, a similar increase in both parameters was observed. While flexibility declined with age, performance on core strength tests and sprint times improved; jumping performance did not change for any gender. In general, there was a positive trend in performance, despite a slight increase in BMI levels, reflecting a positive influence of lifestyle and environmental changes over the past 20 years [25].

In the longitudinal study conducted in Croatia, the main objective was to examine secular trends in health-related physical fitness among Croatian youths between the ages of 7 and 14 between 1999 and 2014. Physical performance was evaluated including body mass index, lower body power measurement, general strength, coordination and agility measurement, upper body strength, flexibility, and cardiorespiratory fitness measurement. While lower body flexibility, power, and cardiorespiratory ability declined in boys, body mass index, upper body strength, and coordination/agility increased. Conversely, in girls, there was an increase in body size, lower body power, upper body strength, coordination/agility, and flexibility; cardiorespiratory appearance decreased, indicating that while upper body strength increased in both sexes, cardiorespiratory fitness decreased in girls [28]. In line with the previous studies taken into consideration, there is a worsening of flexibility levels, which is greater in males, and a decrease in cardiorespiratory endurance capacity linked also in this case to the increase in BMI. In general, physical activity reduced with increasing age in both genders but decreased more among girls. This data may be associated with the earlier biological maturation of girls in comparison with boys. Furthermore, it has been seen that participation in physical activity and organised sports seems to be lower among girls than among boys, probably due to gender stereotypes; therefore, the elimination of factors leading to gender inequality and discrimination in sport might be necessary to increase physical activity among girls [50].

In general, the comparing studies that have assessed strength using BMI sometimes produced conflicting results. While a negative relationship between the variable BMI and the variables aerobic endurance, explosive strength, and motor coordination is evident, the same cannot be claimed for the relationship between BMI and upper body and core strength. This is because the results of this report in the literature are discordant. In fact, some studies state that obese children have more strength in the triceps, quadriceps, and abdominal muscles than overweight and normal-weight children [51]. In fact, in the longitudinal studies examined, core and upper body strength appear to have improved over the years in Portugal and Croatia, while, in Lithuania, the trend is negative. The variability of this relationship underscores the need to further investigate these variables by analysing other related factors as well. 

Sedentary behaviour associated with overweight and obesity was also the subject of the Czech Republic’s longitudinal analysis. The study assessed secular levels and trends of the physical activity and sedentary behaviour of teenagers aged 14 to 18, with the aim of exploring secular trends from 1998 to 2000 and 2008 to 2010 by measuring steps. The results showed no meaningful change in the percentage of overweight teenagers but a significant increase in the proportion of obese teenagers. Over 10 years, the percentage of boys who followed the advice of 11,000 daily steps decreased from 68% to 55%. In contrast, about 74–75% of the girls followed the advice of 9000 daily steps [30].

A later study by Sigmundova and colleagues [52] showed a prevalence of obesity and overweight in both boys and girls, with the exception of 13-year-old girls. After 2006, there was a minor decrease in the reported prevalence of overweight and obese teenagers.

Children and adolescents have many opportunities to be physically active at home and at school, according to the second edition of the Czech Republic Report Card. A considerable proportion of active commuters, high levels of engagement in both organised and unorganised physical activities, and adequate levels of sleep health and physical literacy were also observed. Although there are ample opportunities for children and adolescents in the Czech Republic to participate in physical activity, a notable portion of them fail to meet the current recommendations for physical activity and engage in excessive sedentary behaviour [53].

A comparison with the East was made through the analysis of two studies selected from the literature to understand if the trends were similar. A Korean study analysed children aged 6 to 18 years assessed from 1968 to 2000 by using running distances from 600 to 1200 m and collecting data on aerobic fitness test results. Large datasets from ministries of education were available, as well as six individual studies, and they demonstrated a gradual decrease in Korean children’s aerobic performance (0.26% per year) between 1968 and 1984. After this period, however, there was an intense decline in results and the rate of decline was greater in boys, while, in general, there was a decline in running performance with an increase in body mass index [31].

The majority of Korean children and young people are not sufficiently active, according to Korea’s 2018 Report Card on Physical Activity in Children and Youth, despite ongoing and changing national and international recommendations for physical activity. For instance, since 2005, the percentage of obese teenagers in Korea has been rising steadily. Obesity prevalence in a 2017 representative sample of Korean youth was 9% in girls and 19% in boys. This figure is worrisome given that physical inactivity is a contributing factor in the steady deterioration of physical and mental health [54]. Therefore, in the Korean population, there is a worsening trend in overweight and obesity levels that has been increasing over the years, with direct repercussions on aerobic performance.

Another interesting study conducted in China analysed the anthropometric measurements and physical performance of 12-year-old children living in rural and urban areas, with the aim of investigating trends between 1985 and 2014. The results revealed that the height and weight of both urban and rural children increased over the study period. Furthermore, urban children were found to be taller and heavier than their rural counterparts. Children from urban areas performed better in the fitness tests examined, such as long jumping and running, confirming a general decline in fitness in both urban and rural children after 2000 [23].

The results of a more recent study in Xinjiang, China, show that fitness levels reached their peak in 1995, followed by continued declines in 2005 and 2014. Although this study is out of the current research period, the study shows that physical fitness levels have declined since 2000 [55].

When the PA levels of children in rural and urban areas were compared in a subsequent study conducted in 2021, it was found that a significantly greater proportion of children in rural areas chose to watch TV or use electronic devices than children in urban areas. This is probably because rural communities tend to have fewer adult supervision or family rules, as well as a limited extracurricular lifestyle [56].

The negative trend in Korea and China converges with the Western trend in which physical fitness levels are decreasing. A Canadian study was analysed. In order to study the evolution of various parameters, including flexibility, body composition, muscle strength and power, and cardiorespiratory fitness, a group of researchers sought to offer an overview of the physical fitness of children and young adults between the ages of 6 and 19 years. Fitness measures, assessed by physical fitness tests, changed in the three cycles (2007–2009, 2009–2011, 2016–2017) by age group and gender. There were statistically significant modifications between cycles. Decreases in cardiorespiratory fitness were observed for boys aged 8 to 14 years. Grip strength decreased in boys aged 11 to 19 years. Flexibility remained stable over time, with an improvement observed in girls aged 6 to 10 years [24]. Comparing the Canadian Health Measures Survey (CHMS) with the Canadian Fitness Survey of 1981 reveals a significant decline in the fitness levels of children and young people since 1981, irrespective of age or gender, especially between the ages of 6 and 19. The CHMS is a survey on health measures that was conducted on a representative national sample from 2007 to 2009 and provides descriptive statistics for indicators of body composition, aerobic function, and musculoskeletal function that are analysed by sex and age. The aerobic fitness of young people between the ages of 15 and 19 is generally higher [57].

Confirming these positive data, the Canadian 2022 Report Card on physical activity for young people and children shows that only 28% of children in Canada (5–17 years) meet the national guidelines on physical activity. It also highlights that 25% of children and young people have reached more than 840 min per week (>2 h per day on average) of total time involved in unstructured outdoor and indoor games. 

On the other hand, the trend on the American front regarding the amount of daily physical activity appears to be the opposite. In the USA, research on active commuting, outdoor play, high school sports, and physical education was conducted to study trends in sedentary behaviour and physical activity among young Americans. According to data from the Youth Risk Behaviour Surveillance System (YRBSS) and other national studies and longitudinal surveys in the fields of education, media, security and transport, results vary between age groups. While high school girls’ participation in sport increased from 1971 to 2012, active commuting, physical education in high school, and participation in outdoor games decreased over time. Furthermore, during the first ten years of the twenty-first century, there was an increase in computer use and electronic entertainment [27].

National results collected through National Health and Nutrition Examination Surveys 2001–2004 were analysed by a study that confirmed the trend towards sedentariness [58]. These descriptive analyses reported the percentage of US children (aged between 4 and 11 years) by gender, age, weight, and ethnicity, highlighting low levels of active play (active play was defined as activity of an intensity that causes breathlessness and sweating) and high levels of time spent in front of a screen.

From the longitudinal studies just discussed, one overriding interest that emerges is common to all countries: monitoring levels of participation in physical activity to counter the growing trend towards sedentary behaviour in young people that is inevitably associated with social changes in daily lifestyles.

This work aimed to conduct a literature review on the general trends of several elements of physical activity, with studies starting in the 1970s that adopted a longitudinal approach.

The scientific landscape has repeatedly indicated that continuous monitoring of physical activity levels is essential for the early detection of negative trends, allowing for early intervention to reverse negative inclinations [29], and the scientific literature has focused repeatedly on how physical fitness is an important indicator of health, emphasising the need for meaningful physical fitness and accurate evaluations in young people [59].

The framework that emerges is certainly complex and trends do not always show similarities; sometimes, the data even diverge from the scientific literature. Research focusing on the coordination of school-age children seems to show a sharp deterioration in the quality and quantity of movement skills in school-age children, associated with a general decline in levels of daily physical activity in childhood [60,61], but further data are needed to identify a decisive trend [62].

From the analysis of the selected studies, the relationship between physical activity levels and BMI certainly emerges as the first evident fact: a progressive decrease in physical activity levels and the development of motor performance associated with being overweight was highlighted. High BMI values are associated with lower motor performance and reduced motor skills development, with more significant differences in boys than in girls [24], especially in the age group 13–14 years, that is, in the transition from lower secondary school to upper secondary school [63].

The benefits deriving from regular training and correct lifestyles concern not only the prevention of overweight and obesity [64] but also improving the functionality of the cardiovascular system [65], and have important positive effects on the state of the psycho-physical well-being of young people [66], stimulating processes of cognitive and emotional development [67,68,69]. A second fact that emerges from the comparison between the different surveys examined is the general decline in aerobic endurance levels, always correlated with an increase in BMI and a sedentary lifestyle.

What clearly emerges from the studies is a general tendency, especially on the part of teenagers, to not comply with recommendations regarding physical activity guidelines; in countries where actions to promote and raise awareness of physical activity have been activated, there are slight improvements, at least in the increase in daily minutes dedicated to physical activity [27].

Further investment at both the national and international levels is imperative to enhance and facilitate participation in physical activity among children and teenagers. This investment is crucial for reducing the future health burden associated with inactivity [70].

## 5. Conclusions

Through the analysis of longitudinal studies conducted in European and non-European countries, a summary framework of what has emerged from the scientific evidence has been outlined. The final balance appears to be very complex to define due to the many and varied relationships between the variables examined and at the same time due to the lack of univocal choices made by the various countries in selecting the aspects to be investigated, which are obviously linked to the specificities of the various cultural contexts and thus to the priorities or questions that the individual country considers important to investigate.

To state clearly whether overall we are observing a decline or, on the contrary, an improvement in levels of participation in physical activity or in motor skills and abilities, would be rash for a number of reasons.

The reference age range was very wide and the periods analysed in the studies did not always match each other; the variables examined were not always the same as the cycles and periods that characterised the investigations; gender differences were only considered for a few studies.

Despite the critical points found, what emerges clearly in all the longitudinal surveys is the negative trend in BMI, which is increasing in all the countries analysed, and in cardiorespiratory fitness, which, in close relation to the increase in overweight levels, is in decline. If an unambiguous trend can be declared on the variables just mentioned, it is not possible to declare the same trend for coordination and flexibility, as the evaluations analysed show discordant results between the different countries. The same heterogeneity of results can be seen in the comparison of upper and lower body strength and core strength, the values of which vary when comparing countries, and in the quantity of daily physical activity, the improvement of which is found only in the USA, but without leading to any improvement in BMI.

In conclusion, the literature analysis revealed that the problem of sedentary behaviour is changing significantly in the modern era. In fact, the rate at which technological advancements and our daily lives are becoming more automated and mechanised points to a rise in the number of sedentary children and, as a result, a tendency towards the involution of motor skills and abilities. The pandemic situation may have worsened the trend. It would be interesting after the recovery of normal everyday life to compare the trend that has marked the pre-pandemic period with levels of physical efficiency and participation in physical activities recorded in different countries five or more years after the pandemic. This is also to understand whether the advent of the pandemic that inevitably forced children and young people to decrease motor and sports practice has consequently prompted policymakers to concretise actions aimed at the promotion, dissemination, and implementation of physical activities in different forms in youth.

The implementation of quarantine regulations in numerous nations initiated a scientific focus on examining the behavioural traits of youth during social distancing on a population scale. Research indicates that the imposition of limitations led to a rise in sedentary behaviour among adults as well as a decline in physical activity. Similarly, studies on participation in physical activity programmes at developmental ages during the transition to distance learning showed negative behavioural changes in many countries [71]. UNESCO [72], with its Fit for Life programme, designed to accelerate recovery from COVID-19 and support the development of inclusive policies using sports interventions, with the aim of improving the well-being of the young population.

The motor area is closely connected to other domains of human behaviour. Recent neuroscientific studies have emphasised the motor component of learning that, from a non-linear perspective, allows us to comprehend, by the research on physical behaviour and targeted actions, aspects such as affectivity and relationship, behaviour, and cognition [4].

This study provides a synthetic framework of trends in the evolution of levels of physical and motor fitness (highlighting decline or improvement) in young people that allows us to understand in the different territorial realities what training needs are emerging in light of the existing different cultural situations; this is useful not only to understand, in the light of studies conducted in other countries, where there is a need for further research in this field through longitudinal studies but also how to take action at several levels on the design and implementation of effective improvement plans and learning actions able to promote the health and well-being of young people, starting from childhood.

The development of interventions favouring healthy lifestyles in different formal and non-formal learning contexts (schools, workplaces, local communities/cities, social and health systems) has as its objective both that of preventing the main behavioural risk factors (smoking, alcohol, incorrect diet, and sedentary lifestyle) and that of increasing population coverage in relation to protective factors. Health promotion is not the exclusive responsibility of the health sector but a co-responsibility between institutional and non-institutional bodies for the planning of increasingly shared, widespread actions and interventions which, from a systemic perspective, can act on the individual and the community, both in horizontal and longitudinal continuity to ensure a better quality of life from a lifelong learning perspective, with specific interventions, starting from the developmental age.

## Figures and Tables

**Table 1:** Summary of the literature analysis.

Author	Title	Participants	Aims	Methodology	Main Results	Country	Trend	
Ao, D. et al. [23]	Trends in Physical Fitness Among 12-Year-Old Children in Urban and Rural Areas During the Social Transformation Period in China	N = 136,539: 34,238 (in 1985); 11,664 (in 1991); 17,485 (in 1995); 18,057 (in 2000); 19,254 (in 2005); 17,962 (in 2010); and 17,906 (in 2014). Aged 12.	The study examines trends spanning 29 years (1985–2014) in body size and performance within physical fitness tests among 12-year-old Chinese children residing in both urban and rural areas.	The analysis draws on data obtained from seven cross-sectional surveys conducted as part of the China National Survey of Student Body Constitution and Health. These surveys encompass anthropometric measurements and assessments of physical performance among 12-year-old Chinese children residing in both rural and urban areas.	The physical fitness levels of children in both urban and rural areas have shown a consistent decline since the year 2000.	China	Physical fitness	ꜜ
Colley, et al. [24]	Trends in physical fitness among Canadian children and young people	N = 6284: 2081 (2007–2009), 2133 (2009–2011), 2070 (2016–2017). Aged 6–19 in 10– year period.	To examine the course of time of fitness.	Tests for measurements of body composition, muscle strength and power, flexibility, and cardiorespiratory fitness.	Physical fitness measurements varied across three cycles (2007–2009, 2009–2011, and 2016–2017) by gender and age group. Significant differences were found between cycles. There have been declines in cardiorespiratory fitness among boys aged 8 to 14 years. Additionally, grip strength has decreased in boys aged 11 to 19 years. However, flexibility remained relatively stable over time, with a slight improvement noted in girls aged 6 to 10 years.	Canada	Cardiorespiratory fitness and grip strength	ꜜ
							Flexibility	=ꜛ
Costa, et al. [25]	Secular Trends in Anthropometrics and Physical Fitness of Young Portuguese School-Aged Children	N = 1819 students, (881 males and 938 females). Age = 10 and 11 years old. Evaluation = during their 5th and 6th scholar grades throughout the entire 20-year timeframe.	Objective: to assess Portuguese children’s physical fitness and secular trends in anthropometrics.	An ANCOVA (Analysis of Covariance) model was employed to examine the variability in anthropometric measures (weight, height, and body mass index) and physical fitness indicators (horizontal jump, curl-up, sprint times, sit and reach) over four consecutive five-year periods (1993–1998; 1998–2003; 2003–2008; 2008–2013).	Throughout the past 20 years, heavier boys and more obese girls were present in the 5th and 6th grades. There was a presence of taller girls (up to the third grade). In tests of core strength and sprint times, both boys and girls did well, but as the years went by, flexibility declined. Average jumping ability remained unchanged for both sexes.	Portugal	Core strength test and sprint times	ꜛ
							Body composition	ꜜ
							Flexibility	ꜜ
							Jumping performance	=
Filippone, et al. [26]	Secular trend of involution of motor skills in school age	N = 1137 students (586 males and 551 females) of the secondary school of the 1st degree of Bolzano, which was assessed in the 1st class in 1989–2004.	To verify the existence of centuries-old trends of involution of motor skills.	Longitudinal–cross-sectional study	Secular trend of involution of aerobic resistance associated with an involution of coordinating performance. Secular trend of involution of aerobic endurance associated with involution of co-ordinative performance.	Italy	Aerobic resistance	ꜜ
							Coordination performance	ꜜ
Iannotti and Wang, [27]	Trends in Physical Activity, Sedentary Behavior, Diet, and BMI Among US teenagers, 2001–2009	N = total of 479,674 students; 49% male; aged 11 years (N = 156,383); 13 years (N = 163,729); 15 years (N = 159,562).	To examine 8-year trends in these behaviours in US teenagers ages 11 to 16.	The Health Behaviour Survey of School-Age Children was administered every four years, with the same questions assessing BMI, physical activity, sedentary behaviour, and eating behaviour at each grade level. Logistic and linear regression analysis controlling for age, gender, race/ethnicity, and family affluence were conducted to account for sampling design.	Significant increments were identified in the number of days with at least 60 min of PA, daily fruit and vegetable consumption, breakfast on weekdays and weekends, and BMI.	USA	Daily minutes PA	ꜛ
							Body composition	ꜜ
Kasović, et al. [28]	Secular trends in health-related physical fitness among 11–14-year-old Croatian children and teenagers from 1999 to 2014	N = 5077 children (50.8% female) and teenagers between the ages of 11 and 14 were recruited by five elementary schools.	Investigating secular trends in 7–14-year-old Croatian children and teenagers from 1999 and 2014 of health-related physical fitness	Cross-sectional study	Boys outperformed girls in all physical fitness tests except for the sit and reach test. Between 1999 and 2014, boys experienced increases in body size, upper body strength, and coordination/agility while observing declines in flexibility, lower body power, and cardiorespiratory fitness. Conversely, during the same period, girls demonstrated increases in body size, lower body power, upper body strength, coordination/agility, and flexibility, but showed a decrease in cardiorespiratory fitness.	Croatia	Physical fitness	ꜛ
							Sit and reach test	ꜜ
							Boys: Body size	ꜜ
							Upper body strength and coordination/agility	ꜛ
							Cardiorespiratory fitness	ꜜ
							Girls: Body size	ꜜ
							Lower body power, upper body strength, coordination/agility, and flexibility	ꜛ
							Cardiorespiratory fitness	ꜜ
Radulovic, et al. [29]	Secular trends in physical fitness of Slovenian boys and girls aged 7 to 15 years from 1989 to 2019: a population-based study.	N = 4,256,930; study period: 1989–2019.	To assess the trend of multiple components of physical fitness in children and young people during the entire educational cycle.	Population-based study: the SLOfit test battery consists of the following anthropometric measurements and fitness tests: BMI, triceps subcutaneous fat, 60 s abdominal crunches, 600-metre run, stand-and-reach, bent arm push-ups (BAHs), standing long jump, 60-metre run, backwards hurdle run, and 20 s arm plateau beat.	Increases in body mass index and skin triceps were recorded in all age groups and both sexes. A decline in cardiorespiratory efficiency was observed in each age group until the last ten years.	Slovenia	Body composition and triceps skinfold	ꜜ
							Cardiorespiratory fitness	ꜜ
Sigmundová, et al. [30]	Secular trends: a ten-year comparison of the amount and type of physical activity and inactivity of random samples of teenagers in the Czech Republic	N = 1573; random samples of teenagers (aged 14–18 years).	To estimate the trends and levels of adolescents’ PA and sedentary behaviour in the Czech Republic. To investigate the secular trends from 1998–2000 to 2008–2010 of PA by a pedometer tool and sedentary behaviour.	Two cross-sectional cohorts of adolescents after one decade. Data were collected through weekly monitoring of adolescents’ PA in 1998–2000 and 2008–2010. In the reference sample, an increase in overweight and obesity was found from 5.5% (oldest cohort, 1998–2000) to 10.4% (youngest cohort).	Overweight and obesity among Czech teenagers in the study increased from 5.5% (older cohort, 1998–2000) to 10.4% (younger cohort, 2008–2010). For boys, there were no relevant variations in the total quantity of sedentary behaviour between the cohorts. For girls, the total amount of sedentary behaviour increased significantly for the older cohort (1998–2000) in comparison with the younger cohort (2008–2010).	Czech Republic	Daily PA	ꜜ
							Body composition	ꜜ
Tomkinson, et al. [31]	Secular Trends in the Aerobic Fitness Test Performance and Body Mass Index of Korean Children and Teenagers (1968–2000)	N = 22,127,265 children aged 6–18 years.	Data from 600–1200 m distance running tests conducted between 1968 and 2000 by Koreans aged 6–18 years were analysed.	Three main methods. (1) The authors personally contacted the Korean Ministry of Education and the Ministry of Culture and Tourism to request relevant published and unpublished works. (2) All relevant references in the studies were followed when obtaining published reports. (3) Online research bibliographic databases and the Korean National Sports University library catalogue, using keywords fitness, performance, endurance, distance run, and aerobic.	Between 1968 and 1984, there was a gradual decline in aerobic capacity among Korean children, with a decrease averaging rate of 0.26% per year. However, after 1984, this decline accelerated significantly, averaging 0.80% per year. Moreover, the rate of decline was found to be higher among boys, younger children, and those residing outside the capital city of Seoul. Changes in running ability among Korean children exhibited a similar pattern to the alterations observed in estimated body mass index. Korean children’s performance on aerobic fitness tests sharply declined in comparison to children from other countries, a trend that coincided with an increase in estimated body mass index among Korean children.	Korea	Aerobic performance	ꜜ
							Body composition	ꜜ
							Running performance	ꜜ
Venckunas, et al. [32]	Secular trends in physical fitness and body size in Lithuanian children and teenagers between 1992 and 2012	N = 16,199 (8131 females, 8068 males); 5775 in 1992, 5325 in 2002, 5099 in 2012; aged 11–18 years.	To assess in Lithuanian schoolchildren between 1992 and 2002 and 2002 and 2012 the general decline in physical fitness.	The study used a Eurofit test battery to evaluate balance, flexibility, strength and muscle power, agility, and cardiorespiratory shape. Anthropometry (BMI) calculated.	There was a reduction observed in upper body strength, leg muscle power, flexibility, and cardiorespiratory fitness between the years, 1992 and 2012. Over the same time period, balance improved in both genders, boys’ agility increased, and girls’ abdominal muscle strength increased. Between 2002 and 2012, the detrimental trends in fitness-related aspects that had been noticed between 1992 and 2002 continued. While the improvement in balance continued at a faster pace, the previously observed positive trends in agility and abdominal muscle strength, noted before 2002, either regressed or reversed between 2002 and 2012.	Lithuania	Flexibility, leg muscle power, upper body strength, and cardiorespiratory fitness	ꜜ
							Abdominal muscle strength	ꜜ
							Balance	ꜛ

= stable.

## Data Availability

Not applicable.
